# Defining the rural generalist psychologist: the development and validation of a model of rural psychology practice in Australia

**DOI:** 10.1080/00049530.2026.2739969

**Published:** 2026-10-04

**Authors:** Margot P. Moody, Natasha M. Loi, Adam J. Rock, Kim J. Usher, Kylie Rice

**Affiliations:** a School of Psychology, University of New England, Armidale, Australia; b School of Health, University of New England, Armidale, Australia

**Keywords:** Rural generalist, psychologist, competency framework, rural mental health, services

## Abstract

**Objective:**

The current study aimed to refine and validate a novel model of rural psychology practice in Australia to better support and develop the rural psychology workforce. A secondary aim was to explore participants’ perspectives of the need and utility of the proposed model in practice.

**Method:**

A two-round Delphi survey was conducted, via Qualtrics, with Australian psychologists experienced in rural practice. A total of 28 participants completed Round 1 and 22 participants participated in Round 2. Reflexive content analysis was used to explore Round 1 participants’ textual responses related to model utility.

**Results:**

Consensus was reached after two rounds, resulting in a validated model of rural psychology practice containing nine domains of competence. An overarching domain of *The Rural Context* was foundational to the remaining eight domains: *Clinical Skills, Networking, Cultural Responsiveness, Practitioner Wellbeing, Ethics, Risk, Professional Development* and *Digital Adaptations*. Participants endorsed the need for a model and provided various uses for implementation.

**Conclusions:**

The current study provides an empirically validated model of rural psychology practice. Utility and clinical implications are discussed, and the model aims to improve mental health service development for rural Australians. International implications are discussed.

## KEY POINTS

**What is already known about this topic:**

1. Rural communities experience poorer mental health outcomes and have less access to mental health services, including psychologists, compared to urban communities
2. Psychologists require specific skills, knowledge, and understanding to effectively work in rural practice
3. Other health disciplines, including nursing, medical and allied health, currently utilise competency and skills frameworks to support the education and development of the rural health workforce, such as rural generalists

**What this topic adds:**

1. A framework for rural psychology practice is needed to better prepare and support psychologists to work in rural areas
2. Rural psychologists and relevant stakeholders now have access to the validated model of rural psychology practice included in this paper
3. Such a model has the potential to offer support for rural psychologists reflecting on their practice, serve as a workforce development tool, and promote safe and effective care for rural consumers

The accessibility of mental health services in rural Australia is impacted by an interplay of geographical, individual, and availability challenges (Moody et al., 2026a). Rural Australians would like greater access to services, with practitioners who understand their unique cultural context (Moody et al., 2025). However, there is a scarcity of mental health clinicians and services in rural communities, and rural Australians experience greater health disparities and poorer mental health outcomes than their urban counterparts (Australian Institute of Health and Welfare, 2024b). To better meet service need, the number of mental health clinicians, specifically psychologists, in these communities needs to increase (AIHW, 2024b). However, clinicians first need to be prepared for the contextual and cultural landscape of rural practice (Moody et al., 2026c).

## The rural context

The broader Australian landscape is made up of vast geographic distances and disparate population distributions (Australian Bureau of Statistics, 2026). The *Australian Statistical Geography Standard (ASGS) Remoteness Structure* classifies rurality according to major cities, inner regional, outer regional, remote, and very remote areas (Australian Bureau of Statistics, 2023). For the purposes of the present study, the term “rural” is used to refer to all areas outside of major cities, unless otherwise specified. To this end, rural regions are characterised by small communities with unique social, ecological, and cultural backgrounds (Lawrence-Bourne et al., 2020). Adversities experienced by rural residents, such as health and financial disparities, are often impacted by the intersectionality of rural communities, in that individual, community, and societal challenges tend to overlap (Lawrence-Bourne et al., 2020). Furthermore, each community carries its own distinct socio-cultural identity, impacted by the economic drivers and historical background of the region (Bischoff et al., 2014). The coalescence of these features in rural communities have implications for health service availability and accessibility.

The isolation of rural Australia can make it difficult to establish cost effective and accessible services (Manning, 2010). Though telehealth has been cited as a solution to service shortages (Bradford et al., 2016), infrastructure challenges, digital literacy, and clinicians with limited contextual understanding can impact users’ engagement with online services (Alam et al., 2019; Fisher et al., 2024; Moody et al., 2025). Furthermore, the rural mental health workforce is comparatively small to the urban workforce (Australian Institute of Health and Welfare, 2024a). Clinicians who choose to enter rural practice face a myriad of unique challenges such as personal and professional isolation, high workloads, and cultural and ethical issues (Helbok et al., 2006; Moody et al., 2026b; Sutherland & Chur-Hansen, 2014). Such challenges can lead to an increased risk of burnout, impacting clinicians’ longevity in rural practice and leaving rural communities unsupported (Cosgrave et al., 2015).

According to Katzenellenbogen et al. (2013) rural pipeline model, a rural workforce framework applicable to all health disciplines throughout a clinician’s training, a number of strategies can encourage a career in rural health, such as selecting rural students into university programmes, teaching rural content, or providing rural placements. This model posits that it is easier to address modifiable factors influencing recruitment and retention (e.g., education) than personal or lifestyle factors, with the authors advocating for greater rural specific training in order to grow the rural health workforce and support practitioners long term (Katzenellenbogen et al., 2013). Amongst general practitioners (GPs), rural experience during undergraduate and postgraduate training, particularly longer placements or those within rural-focused curricula, has been found to positively predict future rural practice (Ogden et al., 2020). Likewise, for both nursing and allied health disciplines, rural training programmes have found to be associated with an increased likelihood of future rural practice work (Brown et al., 2017; Playford et al., 2010). For example, a review of a rural-based tertiary education programme for speech and language therapists found that most students continued working in rural practice and reported intentions to remain there (Smith-Tamaray et al., 2021). However, although some postgraduate psychology programmes have utilised rural-based placements, the majority take place in urban settings (Little et al., 2019). Rural-based placements can be difficult to source and implement due to the limited number of supervisors and systemic support available to students in rural communities (Little et al., 2019). Likewise, within university programmes, rural psychology content is limited. In Australia, only one tertiary programme relevant to rural psychology practice is currently available, by way of the Rural Generalist Program delivered via James Cook University (2025). However, entry into the programme is open to the broader allied health discipline, with cadre-specific content minimal (James Cook University, 2025).

## Rural health competencies

For some health professions, such as medicine and nursing, rural practice is recognised as a specialised field, allowing for targeted training, recruitment, and support (Office of the National Rural Health Commissioner, 2023; Worley et al., 2019). These fields have also utilised competency frameworks to assist in describing the components of rural health practice and distinguish it from core therapeutic skills (Barker et al., 2021; Office of the National Rural Health Commissioner, 2023; Worley et al., 2019). A competency model can be understood as a “framework for defining the skills and knowledge requirements of an occupation” (von Treuer & Reynolds, 2017, p. 2). In establishing rural specific competencies, health professions can recognise the uniqueness of rural health practice and train clinicians at all levels of the rural health pipeline. Likewise, as well as informing relevant tertiary programmes, rural competency models can be useful for recruitment, performance reviews, and assisting in identifying professional development needs throughout a clinician’s career (Lin et al., 2009). The medical profession has long recognised the rural practitioner, most recently by way of the national rural generalist programme. This programme has allowed for specialised training pathways and incentivised programmes aimed at increasing the rural health workforce (Department of Health, Disability, and Ageing, 2025).

For allied health practitioners, the Allied Health Rural Generalist (AHRG) Education Framework aims to directly support the training of rural practitioners by way of the AHRG Pathway (Queensland Health, 2018). This framework consists of four domains of practice including rural generalist service delivery, rural and remote practice, profession-specific clinical practice (e.g., psychology), and service-specific clinical skills (Queensland Health, 2018). Research into its accompanying tertiary programme at James Cook University (2025) has demonstrated benefits to participating practitioners, employers, relevant organisations, and consumers by supporting clinicians to improve their evidence-based rural generalist skills and knowledge (Dymmott et al., 2024). However, relevancy of both the AHRG framework and tertiary programme to specific disciplines and contexts remains an important consideration. Overall participation in the programme by psychologists is low (Dymmott et al., 2024), and research is yet to examine programme outcomes specific to disciplines. Currently, no such frameworks exist within psychology, despite recognition among clinicians that practicing in rural contexts requires a distinct combination of skills, contextual knowledge, and professional competencies (Moody et al., 2026c; Sutherland & Chur-Hansen, 2014).

In Australia, all psychologists are required to abide by professional practice standards as part of their annual registration (Psychology Board of Australia, 2024). These standards outline the core professional competencies and code of conduct required by the psychology profession as prescribed by the Psychology Board of Australia. The Professional Competencies for Psychologists are used throughout a clinician’s training and career to ensure safe and effective practice (Psychology Board of Australia, 2024). Previous research found that competencies and skills used by rural psychologists largely align with these standards of practice; however, the rural context dictates how skills are implemented and utilised (Moody et al., 2026c). For example, though all psychologists strive to minimise multiple relationships, these may be unavoidable in small and isolated communities. Rather, rural psychologists need to be comfortable navigating the increased likelihood of multiple relationships while maintaining ethical, safe, and professional standards of practice (Moody et al., 2026c). Currently, rural psychologists operate with limited support and without guidelines to help translate professional practice standards to the unique challenges of rural settings.

## The present study

To better support the rural psychology workforce and improve mental health service access, a rural psychology framework is needed. Therefore, the current study developed an initial model and addressed the following research questions: (1) Can a novel model of rural psychology practice achieve expert consensus and validation using the Delphi method? And (2) What are rural psychologists’ perceptions regarding the need and utility of such a model?

## Method

### Model development

In response to the need for greater clarity regarding the role of rural psychologists, a novel model was developed by the authors, outlining the knowledge, skills, and competencies psychologists require to practice effectively in rural contexts. The model was conceptualised from an integration of consumer perspectives (Moody et al., 2025, 2026a), qualitative research with rural psychologists (Moody et al., 2026b, 2026c), and relevant rural psychology literature (e.g., Australian Psychological Society, 2016; Bischoff et al., 2014; Bourke et al., 2004; Helbok et al., 2006; Sutherland & Chur-Hansen, 2014). Consistent themes and recommendations from the preceding literature were consolidated into an initial framework. Model components were reviewed and refined through an iterative process by the research team throughout development. A substantial portion of the preliminary model development was informed by the authors’ own programme of research (Moody et al., 2025, 2026a, 2026b, 2026c), including one manuscript under review at the time of this manuscript’s submission (Moody et al., 2026b). The present study was designed to provide an independent empirical basis for refining and validating the model. As such, additional research identified through an iterative review of literature relevant to the topic, was incorporated into the model development.

The initial model was also refined through expert review. Two psychologists and researchers with experience in rural and regional areas and independent from the main research team, provided verbal feedback resulting in minor amendments. Following the development phase, the model consisted of an overarching domain of *the rural context*, visually depicted to impact skills and knowledge of a further eight competency domains: *clinical skills, networking, cultural responsiveness, practitioner wellbeing, ethics, risk, professional development*, and *telehealth*. All domains, with the exception of *telehealth*, consisted of multiple items. See Figure 1 for the initial model. When developing a competency model using multiple data sources, such as surveys, subject matter experts (SMEs), and validation techniques, can improve the model’s robustness and increase the likelihood that it will be used in practice (Campion et al., 2011). Therefore, the current study aimed to refine and validate the model via an online survey, utilising the Delphi method, in order to present a valid competency framework to rural psychologists. The Delphi approach was chosen due to its ability to gather iterative, anonymous consensus from geographically dispersed experts (Chalmers & Armour, 2019), such as Australian rural psychologists.

**Figure 1.**
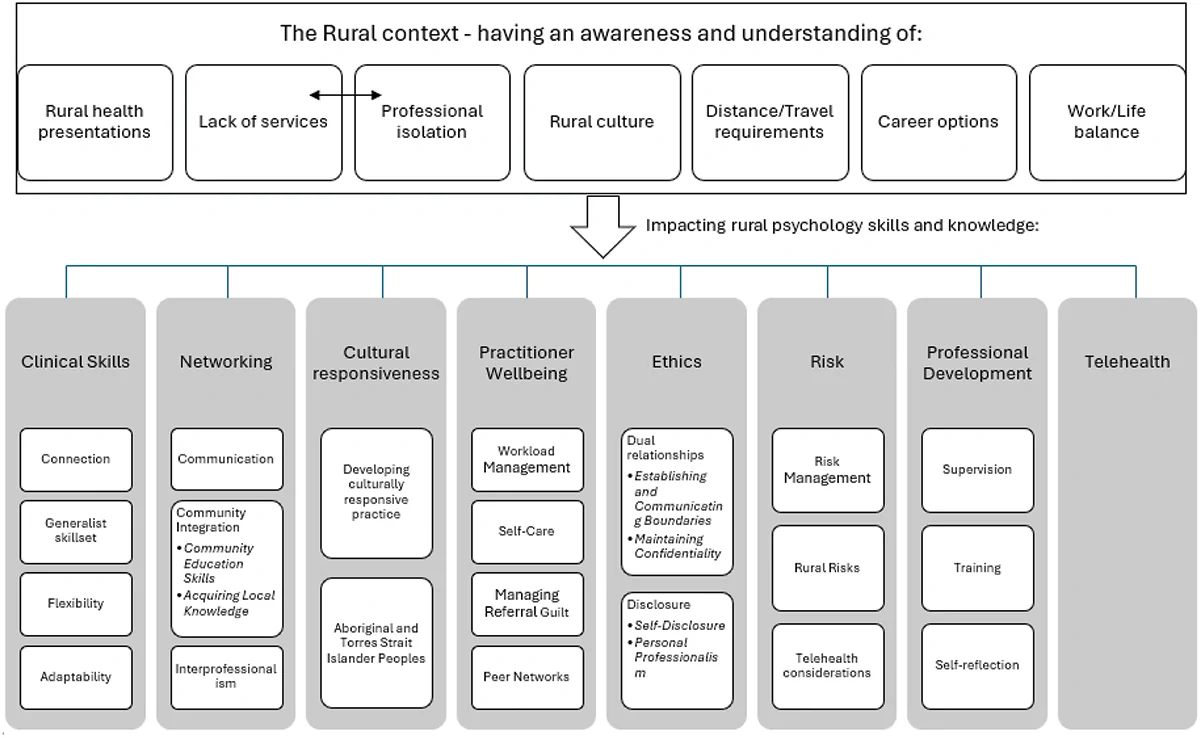
The initial proposed model of rural psychology practice presented to the Delphi panel.

### Participants

Participants were recruited across Australia via professional networks, such as the Australian Psychological Society Rural and Remote Interest Group and local government health services. As per the Delphi process, the use of SMEs is important for the validity of evidence-based competency modelling (Lievens et al., 2004). There is no consensus on how to define experts in Delphi; rather, participants should possess a certain level of knowledge in the area (Chalmers & Armour, 2019). For the current study, SMEs included those with experience in rural psychology, either in a clinical or research capacity, with participants self-identifying relevance to the topic based on the study description and recruitment invitation. The decision to include academic and clinical participants ensured both practical and theoretical perspectives were considered, supporting diverse representation within the sample. A total of 30 participants took part in Round 1 of the survey, with two participants’ responses removed due to incomplete responses. In Delphi studies, the minimum acceptable number of participants is generally above seven (Chalmers & Armour, 2019), with sample sizes varying depending on the study aims and research questions (Schifano & Niederberger, 2025). Considering the research aims and sample heterogeneity, the sample size was deemed acceptable by the research team, while allowing for reasonable participation dropout between Rounds 1 and 2. As two participants did not provide contact information, 26 participants were contacted to participate in Round 2, with 22 completing both rounds.

Of the Round 1 sample, the majority of participants were from a regional location (71.4% inner regional, 17.9% outer regional), with 7.1% from a major city. One participant did not provide their location. The sample included 22 females and six males. Most participants were aged 40–49 (*n* = 9; 32%), followed by 30–39 (*n* = 7; 25%). Twenty-five participants identified as Caucasian, with participants also identifying as Aboriginal and Torres Strait Islander, Middle Eastern, and South Asian. On average, participants had 15 years’ experience in clinical practice or supervision (*SD* = 12), and 3.2 years’ experience in academia (*SD* = 3.9).

### Delphi procedure

Participants took part in a Delphi process, a commonly used technique in health research, particularly in areas where the evidence base is limited, as it enables the integration of available evidence and expert opinion to inform understanding (Chalmers & Armour, 2019). The Delphi is an iterative method relying on expert consensus to evaluate, refine, and converge on the problem solving and decision making of complex ideas (Chalmers & Armour, 2019). For the current study, two rounds were required to reach consensus for all model items. Ethical approval for each survey round was provided by the host University’s Human Research Ethics Committee. At the beginning of each round, participants read an information sheet and provided implied consent. See Figure 2 for a summary of the study procedure. This study was reported with reference to the DELPHISTAR reporting recommendations for Delphi studies (Niederberger et al., 2024).

**Figure 2.**
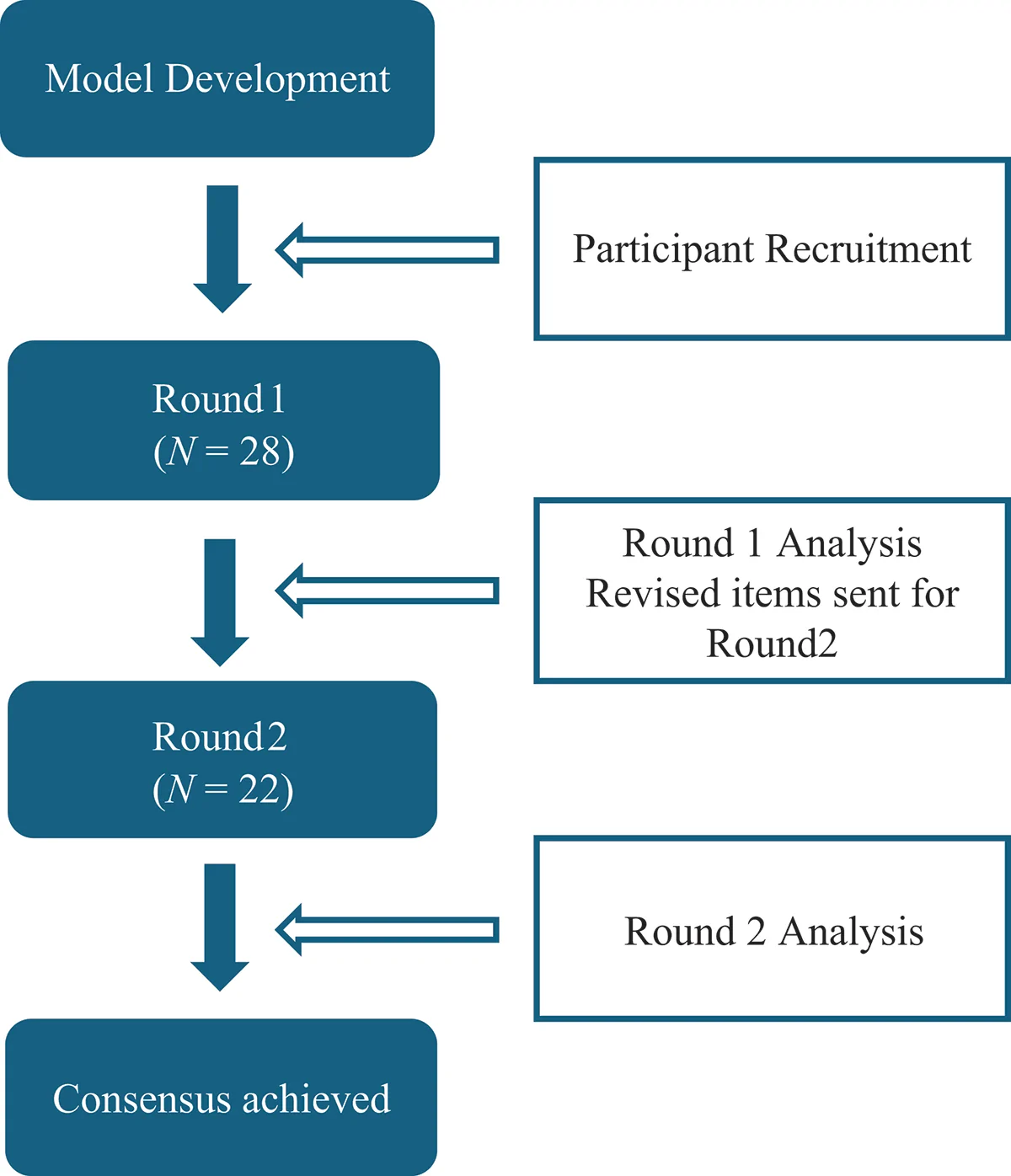
Summary of the study procedure as per the Delphi method.

#### Round 1

For Round 1, participants were asked to respond to demographic items at the beginning of the survey, including providing their postcode, age range, gender, ethnicity, and professional experience. Postcodes were used to class rurality as per the *ASGS Remoteness Structure* (Australian Bureau of Statistics, 2023). Participants were asked to rate the importance of developing a model of rural psychology practice. Responses were scored using a 5-point Likert scale from 1 (*not important at all*) to 5 (*essential)*. Participants were asked to explain their rating via textual response using the open-ended question: *“Why do you believe it [a model of rural psychology practice] is important/not important?”* Participants were then presented with the initial model and asked to indicate their level of agreement with each domain and its associated items. Responses included *yes, no*, or *maybe*, with those who responded *no* or *maybe* encouraged to provide a textual response detailing suggested amendments. All participants were offered the opportunity to share additional feedback throughout the survey. For each domain, brief item-specific descriptions were provided. At the end of the survey, participants were asked to rate their overall agreement with the model. Additional open-ended questions requiring textual responses included participants’ perspectives on the utility of the model for both practitioners and rural communities. Questions included: *“What do you believe would be the impact or benefit (if any) of this model for practice?”* and *“What do you believe would be the impact or benefit (if any) of this model in terms of preparing practitioners to work in rural areas?”*

Following Round 1, participant feedback was consolidated and integrated into a revised model before being reviewed by the research team. Participants were then contacted to complete Round 2, which sought consensus on revised items and proposed amendments to the overall model.

#### Round 2

Survey items for Round 2 were reduced due to the majority of items reaching consensus in Round 1. Round 1 feedback was shared with participants by way of a narrative description of items that reached consensus and items requiring a second review. Participants were again asked to rate their level of agreement with the overall model and items that had undergone major revisions. Following Round 2, consensus was reached and no further rounds were necessary.

### Analysis

#### Consensus

Although there are no established guidelines for consensus in Delphi research, many studies in health research define it as 70–80% agreement (Chalmers & Armour, 2019; Schifano & Niederberger, 2025). For the current study, consensus was defined a priori as 80% agreement, with descriptive statistics used to calculate participant agreement. For all items, including items that reached over 80% agreement, participant feedback was incorporated into model revisions. Items that did not meet consensus, or that underwent major revisions, were presented in Round 2. Using this approach, two rounds were required to reach consensus on all items.

#### Content analysis

Reflexive content analysis (RCA; Nicmanis, 2024) was employed to analyse participants’ textual responses relating to the need and utility of a rural psychology competency model. RCA is used to describe explicit qualitative data using a hierarchical structure to understand patterns in the data (Nicmanis, 2024). As the aim of analysis was to provide a descriptive response in answer to short-answer questions rather than to explore latent meanings or interpretations of participants’ responses, RCA was deemed an appropriate method. Analysis was completed primarily by the first author, following the stages prescribed by Nicmanis (2024). This included familiarisation with the data, generating and revising codes, and creating and reviewing categories. Categories were further reviewed by the research team during reporting stages, with discrepancies discussed until consensus was reached. Following iterative refinement, 16 final codes across all short-answer questions were generated. These codes were then further consolidated into three categories relating to model need and four categories relating to model utility.

Reflexivity is a central tenet of RCA, seen as an analytical tool in continuously exploring and understanding findings (Nicmanis, 2024). To this end, the first author, a researcher and practitioner with training in clinical psychology and experience in rural mental health, utilised this background by engaging in ongoing reflexive practice, acknowledging how professional background and experience interacted with the coding process and category development. Likewise, throughout the analytical process, researcher reflexivity was further supported through continual review of categories and codes and discussions with the wider research team, which included members with expertise in psychology, rural mental health, and qualitative methodology.

## Results

### Round 1

Following Round 1, consensus was reached on all survey items, with the exception of the *telehealth* domain. See Table 1 for percentage agreement for both Rounds 1 and 2. Feedback provided by participants by way of open-ended responses informed major revisions to the *telehealth* domain, renamed as *digital adaptations*, and included the development of four new items: *knowledge of technology, technology as an adjunct for services, risk*, and *rural considerations*. Likewise, a new item, *working with rural communities*, was developed in response to participant feedback regarding the *cultural responsiveness* domain. Additional participant feedback to the remaining domains and items resulted in minor changes to competency descriptions and labels. For example, the item *Aboriginal and Torres Strait Islander peoples* was renamed *working with Aboriginal and Torres Strait Islander peoples*, and *dual relationships* was renamed *multiple relationships*. These changes did not alter item meanings; rather, they focused on improving wording and clarity and, as such, did not require a second review.

**Table 1.** Percentage of participant agreement with model items.

Competency domains	Round 1 (*N* = 28)			Round 2 (*N* = 22)		
	Yes	No	Maybe	Yes	No	Maybe
**The Rural Context**	85.7	0	14.3	–	–	–
Rural health presentations	92.9	0	7.1	–	–	–
Lack of services	100	0	0	–	–	–
Professional isolation	92.9	0	7.1	–	–	–
Rural culture	92.9	0	7.1	–	–	–
Distance/Travel requirements	92.9	3.6	3.6	–	–	–
Career options	89.3	0	10.7	–	–	–
Work/Life balance	82.1	3.6	14.3	–	–	–
**Clinical Skills**	96.4	0	3.6	–	–	–
Connection	82.1	0	17.9	–	–	–
Generalist skillset	92.9	0	7.1	–	–	–
Flexibility	89.3	0	10.7	–	–	–
Adaptability	92.9	0	7.1	–	–	–
**Networking**	96.4	0	3.6	–	–	–
Communication	96.4	0	3.6	–	–	–
Community integration	89.3	0	10.7	–	–	–
*Acquiring local knowledge*	100	0	0	–	–	–
*Community education skills*	96.4	0	3.6	–	–	–
Interprofessionalism	100	0	0	–	–	–
**Cultural Responsiveness**	89.3	0	10.7	–	–	–
Working with rural communities	–	–	–	95.5	0	4.5
Working with Aboriginal and Torres Strait Islander Peoples^a^	85.7	0	14.3	–	–	–
Developing culturally responsive practice	96.4	0	3.6	–	–	–
**Practitioner Wellbeing**	96.4	0	3.6	–	–	–
Workload management	96.4	0	3.6	–	–	–
Self-care	96.4	0	3.6	–	–	–
Managing referral guilt	92.9	0	7.1	–	–	–
Peer networks	100	0	0	–	–	–
**Ethics**	100	0	0	–	–	–
Multiple relationships^a^	96.4	0	3.6	–	–	–
*Establishing and communicating boundaries*	89.3	0	10.7	–	–	–
*Maintaining confidentiality*	96.4	0	3.6	–	–	–
Disclosure	96.4	0	3.6	–	–	–
*Self-disclosure*	85.7	3.6	10.7	–	–	–
*Personal professionalism*	100	0	0	–	–	–
**Risk**	100	0	0	–	–	–
Rural risks	100	0	0	–	–	–
Risk management	96.4	0	3.6	–	–	–
Telehealth considerations^c^	96.4	0	3.6	–	–	–
**Professional Development**	89.3	3.6	7.1	–	–	–
Supervision	92.9	0	7.1	–	–	–
Training	85.7	0	14.3	–	–	–
Self-reflection	89.3	3.6	7.1	–	–	–
**Digital Adaptations**^**b**^	28.6	0	71.4	95.5	0	4.5
Knowledge of technology	–	–	–	90.9	0	9.1
Technology as an adjunct for services	–	–	–	100	0	0
Risk^c^	–	–	–	86.4	4.5	9.1
Rural considerations	–	–	–	95.5	0	4.5
Overall Model	82.1	0	14.3	95.5	0	4.5

Note: Working with rural communities and Digital adaptations appeared in the Round 2 survey.

^a^Wording of items changed throughout rounds. Wording used in the table is the same as the wording used in the final validated model.

^b^Digital Adaptations appeared as Telehealth in Round 1.

^c^Telehealth considerations was renamed Risk, under Digital Adaptations for Round 2.

### Round 2

As per Table 1, all items reached consensus following Round 2, with the overall model achieving 95.2% agreement amongst participants. Similar to Round 1, participant feedback was incorporated into revisions, resulting in minor changes to domain and item descriptions.

### Final model

The final model included the overarching domain *the rural context*, informing a further eight competency domains: *clinical skills, networking, cultural responsiveness, practitioner wellbeing, ethics, risk, professional development*, and *digital adaptations*. See Figure 3 and Table 2 for the visual representation and item descriptions, respectively.

**Figure 3.**
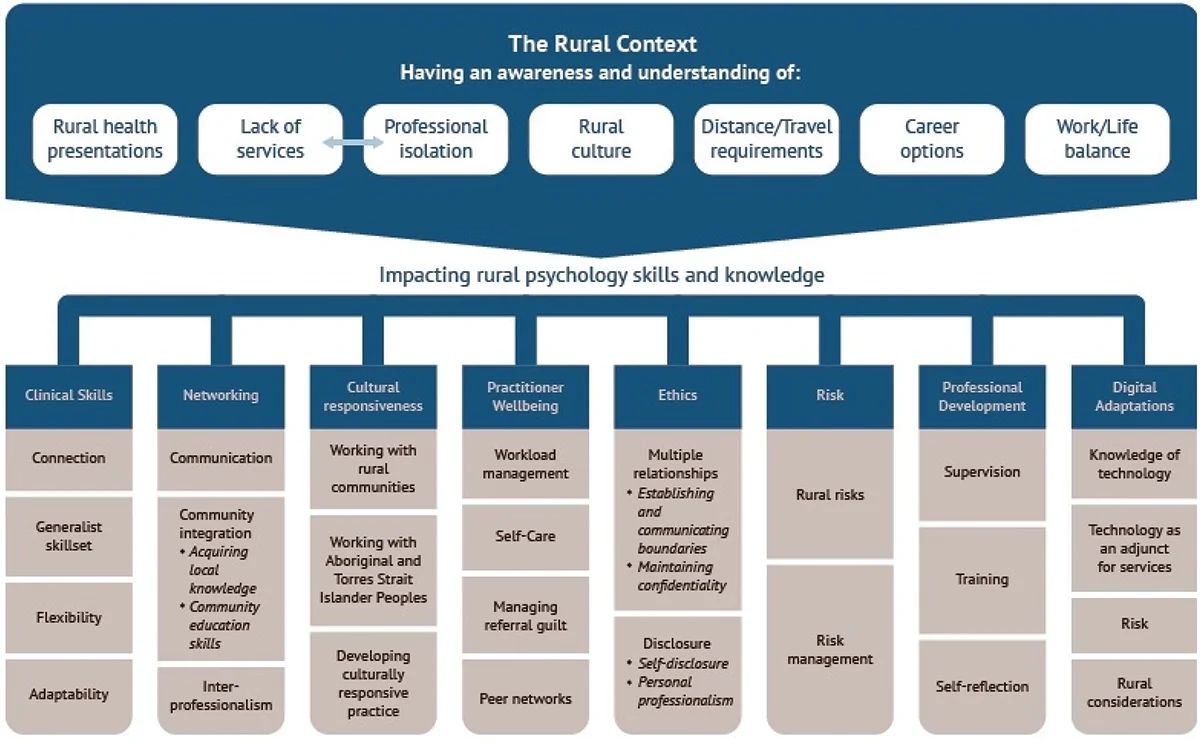
Validated model of rural psychology practice.

**Table 2.** Item descriptions for the model of rural psychology practice.

Competency Domain	Items	Description
1.0 The Rural Context	1.1 Rural Health Presentations	Knowledge of health risk factors in rural areas (e.g., higher frequency of chronic illnesses); understanding how rural factors (e.g., socioeconomic factors, drought) may impact mental health presentations
	1.2 Lack of Services	The limited number of services available in rural areas and impacts on caseload, care coordination, ethical responsibilities, among others. Lack of services can increase feelings of professional isolation (visually linked in model)
	1.3 Professional Isolation	At times, practising as the only psychologist in the team, practice, or community and the impacts of this
	1.4 Distance/Travel Requirements	Travel requirements of rural practice, both personally and professionally (travelling to see clients, access professional development/supervision)
	1.5 Rural Culture	The unique culture of rural communities and understanding that each community carries its own culture and history
	1.6 Career Options	Reflects the duality of providing support to communities that otherwise would not have access to services, which in turn may limit opportunities for specialised roles or career growth
	1.7 Work-Life Balance	Living/working rurally can provide a unique and flexible lifestyle, inclusive of remoteness challenges such as personal isolation and reduced anonymity in communities
2.0 Clinical Skills	2.1 Adaptability	The ability to adapt a particular treatment or modality to best fit with the client (and rural context/environment), while remaining evidence-based and relevant to that person
	2.2 Generalist Skillset	Having a diverse range of skills and knowledge to treat various presentations across the lifespan of differing levels of risk and complexity, while acknowledging limits of competency
	2.3 Flexibility	The ability to flexibly manage scheduling, referrals, or working across roles
	2.4 Connection	Prioritising rapport and building a strong working relationship with diverse clients
3.0 Networking	3.1 Communication	Clear communication skills with individuals, the broader community, and other health professionals in a style relevant to that community
	3.2 Community Integration	Understanding the importance of embedding oneself into the community, either in-person or via distance, and developing personal and professional connections whilst maintaining ethical boundaries
	*3.2.1 Acquiring local knowledge*	Understanding the importance of gaining local knowledge, including relevant local services, historical events and impacts etc.
	*3.2.2 Community education skills*	The ability to deliver psychoeducation to other health professionals, and larger community events
	3.3 Interprofessionalism	The ability to connect and collaborate with other health professionals and community stakeholders relevant to the consistency of care for clients
4.0 Cultural Responsiveness	4.1 Working with Rural Communities	Understanding the unique cultures and identities that underpin every distinct rural community, and delivering culturally and contextually relevant practice tailored for each individual setting
	4.2 Working with Aboriginal and Torres Strait Islander peoples	Knowledge and awareness of Aboriginal and Torres Strait Islander cultures and ability to seek understanding of local cultures/practice
	4.3 Developing Culturally Responsive Practice	Knowledge and awareness of other cultures while acknowledging own biases and impacts to practice
5.0 Practitioner Wellbeing	5.1 Workload Management	Skills managing caseload/schedule to avoid burnout and maintain wellbeing
	5.2 Self-Care	Developing and prioritising self-care practices that are achievable in rural settings
	5.3 Managing Referral Guilt	Awareness of moral challenges regarding accepting/rejecting referrals; developing personal guidelines to manage referrals, including the ability to manage conflicts in contexts where expectations do not match guidelines
	5.4 Peer Networks	Ensuring connectivity with psychology peers
6.0 Ethics	6.1 Multiple Relationships	Awareness of the types of multiple relationships likely in rural communities; developing practices to manage these ethically
	*6.1.1 Establishing and Communicating Boundaries*	Establishing and communicating boundaries when accepting referrals, working with clients, and engaging with the wider community, including re-negotiating boundaries as roles and relationships change
	*6.1.2 Maintaining Confidentiality*	Awareness of psychologists’ multiple roles in the community and maintaining confidentiality across all roles
	6.2 Disclosure	Awareness and understanding of the various types and levels of disclosure that may occur in a rural community
	*6.2.1 Managing Self-disclosure*	Understanding the need to share more of oneself, either incidental or deliberate, to develop trust with clients and the community, while maintaining ethical boundaries
	*6.2.2 Personal Professionalism*	Managing conduct and presence in the community within both one’s personal and professional life. Learning to balance the intersection of personal and professional identity in this context
7.0 Risk	7.1 Rural Risks	Knowledge of how risk may present in rural areas; developing capacity to work with and manage higher risk
	7.2 Risk Management	Adapting risk assessments, safety plans, and crisis intervention to the rural context. Consideration for managing rural specific risks, such as access to firearms and farm equipment, within an individual’s work and lifestyle context
8.0 Professional Development	8.1 Training	Prioritising and maintaining professional development needs relevant to a broader scope of practice. Proactively identify relevant in-person and online training opportunities that are accessible in rural contexts
	8.2 Supervision	Developing the resources and networks to access supervision (both peer and individual) when needed, including remote support
	8.3 Self-reflection	Ongoing engagement in reflective practice, particularly in managing risk, ethical or cultural issues relevant to the rural context
9.0 Digital Adaptations	9.1 Knowledge of Technology	Developing digital literacy, including awareness of e-health interventions, modes of delivery, and digital privacy issues relevant to rural practice
	9.2 Technology as an Adjunct for Services	Adapting interventions to align with the available resources, infrastructure, and capabilities of communities, with the understanding that technology is a necessary adjunct to increase service access in rural areas
	9.3 Risk	Developing practices to ensure safety and manage risk during telehealth calls and e-health interventions
	9.4 Rural Considerations	Applying the rural context to digital mental health, and understanding how this impacts the feasibility of both accessing and delivering services

### Textual responses

Of the 28 participants who took part in Round 1, not all provided responses to open-ended items, and each response could be analysed using multiple codes. Therefore, the number of participants who responded to each question is indicated in Table 3.

**Table 3.** Reflexive content analysis of open-ended responses.

		Preference Rationale (*n* = 26)	
Category	Example Codes	*R*	
*Why is a model of rural psychology practice important?*			
Limited services	Lack of access, Meeting need	12	
The rural context	Practice is different; Population is different	29	
Rural psychologists need support	Training; Challenges of rural practice	9	
		For Practice (*n* = 22)	For Clinicians (*n* = 23)
Category	Example Codes	*R*	*R*
*What do you believe would be the impact or benefit of this model?*			
Self-reflection	Self-assessment tool; reflective; validating	13	6
Workforce development	Rural practice awareness; organisation tool; transition from urban	19	26
Training	Training and supervision; university curriculum	12	8
Effective practice	Protect clients	2	2

Note: *R* represents number of responses provided.

#### Model need

Participants were asked to rate the necessity of a model of rural psychology practice, with 61% of participants indicating a model was very important, 32% of participants indicating a model was essential, and 7% of participants indicating a model was of average importance. When asked to provide their reasoning, analysis of textual responses identified three categories of rationale: *limited services, the rural context is unique*, and *rural psychologists need support. Limited services* referred to the need for more psychologists to practice rurally to improve service access in rural communities, given the lack of mental health services currently available. *The rural context is unique* category was inclusive of both rural practice and the population it services, and how many health, social, cultural, and geographical factors differentiate these contexts from the urban environment. For example, the way in which local infrastructure (access to internet), natural disasters, and multiple relationships can impact the way in which communities seek and receive support. Participants responded that because of these differences, rural psychology practice can be challenging for clinicians and that rural specific training would assist in preparing clinicians to overcome challenges and work well in the unique rural context, as reflected in the *rural psychologists need support* category.

#### Model utility

Participants were further asked to comment on the potential utility of the proposed model for both practice and clinicians. Across both contexts, participants reported four categories of use: *self-reflection, workforce development, training*, and *effective practice*. Under the *self-reflection* category, respondents indicated the model offered utility for current rural practitioners as a self-assessment and reflective practice tool to direct relevant professional development. This process would also validate their roles as a shared and important experience, forming a rural practitioner identity and allowing opportunities for the profession to progress. As one respondent wrote: *“to be able to label the specific seems extremely important for then understanding how we can, both individually and as a collective, continue to move forward and progress as a profession”* (Participant #16). *Workforce development* referred to recruitment and retention use, ensuring psychologists, communities, and other relevant stakeholders were aware of rural practice and its role requirements (*“Raise awareness of the uniqueness of working in these communities”*, Participant #1). Likewise, the model could prepare interested clinicians transition from urban practice or help them decide whether such a role would be an appropriate fit (*“Psychologists considering working in a rural location can better understand the contexts of the role before accepting it”*, Participant #8). Regarding *training*, respondents described how the model could assist with training rural relevant competencies to psychologists through professional development opportunities, as well as university students. Under this category, respondents also highlighted the utility of the model in supporting urban-based supervisors unfamiliar with rural practice: *“Better understanding for supervisors of rural psychologists who may not understand the context of their supervisees”* (Participant #3). Categorised as *effective practice*, a small number of respondents acknowledged that using such a model, and supporting clinicians to develop competencies required for rural practice, can help ensure effective and safe practice for rural communities. As one respondent wrote *“It* [the model] *can act as a guide for competencies and highlight areas that clinicians need to work on. This also would help protect rural and remote clients”* (Participant #14).

## Discussion

The current study aimed to refine and assess a model of rural psychology practice. Following two rounds of Delphi surveys, experts in rural psychology reached consensus on all items, resulting in a validated framework describing the key competencies and skills required to deliver and sustain rural psychology practice. The majority of participants rated the model to be either very important or essential, identifying a variety of potential uses in practice, including as a self-reflective tool, for workforce development, training and supervision, and to ensure effective client care.

The validated model of rural psychology practice consists of nine domains of competence, including an overarching competency domain of *the rural context*. It offers a novel framework of the requirements and skills of rural psychology practice in Australia. Original competence domains and items were sourced from an integration of studies with both rural mental health consumers and psychologists (Moody et al., 2025, 2026a, 2026b, 2026c) as well as relevant prior literature (e.g., Australian Psychological Society, 2016; Bischoff et al., 2014; Bourke et al., 2004; Helbok et al., 2006; Sutherland & Chur-Hansen, 2014), with the majority of items reaching consensus following Round 1. Two domains underwent considerable refinements based on Round 1 feedback: *cultural responsiveness* and *telehealth*. The *cultural responsiveness* domain was strengthened by the creation of the working with rural communities item. Though understanding rural culture was emphasised in the *rural context* domain, participants spoke of the skills necessary to ensure rural knowledge translated into culturally and contextually relevant care. Cultural responsiveness refers to the awareness of the social and cultural factors of a client’s cultural background and engaging in ongoing reflexive practice to improve practice and client outcomes (Psychology Board of Australia, 2024). Participants’ inclusion of the *working with rural communities* item within this domain, further highlights the importance of cultural responsiveness, rather than just cultural competence, in psychology practice.

Similarly, the former *telehealth* domain was expanded into the *digital adaptations* domain. Since the COVID-19 pandemic necessitated a shift away from traditional face-to-face care, the use of digital health technologies has rapidly increased, particularly in rural and remote areas (Krahe et al., 2024). Digital health encompasses any technology used to facilitate and enhance care, including virtual technologies, artificial intelligence, webpages and applications, in addition to telehealth (Krahe et al., 2024, 2025). However, in rural areas, effective implementation of digital health requires strategic management to ensure that these methods are compatible with local and cultural systems (Krahe et al., 2025). Participants’ feedback on the original *telehealth* domain indicated both the broader use of digital health and the need to contextualise its implementation within each rural community. As such, new items were developed to (1) recognise the various ways digital health could support communities, (2) acknowledge the challenges many rural people face when accessing telehealth services (e.g., connectivity, difficulty developing rapport; Fisher et al., 2024), and (3) ensure proposed services were both feasible and accessible to the population clinicians supported.

### Alignment with rural health frameworks

Currently, the only comparable framework available to rural psychologists is the AHRG Education Framework (Queensland Health, 2018). Though the rural psychology model shares some consistencies with the domain units reported in the AHRG Framework, such as telehealth, cultural safety, and ethical practice, the newly developed rural psychology model is unique and specific to the psychology profession. Furthermore, the AHRG Framework was primarily developed to structure and inform relevant rural generalist training programmes, limiting its scope beyond this context. Rather, our newly developed psychology rural practice model offers utility similar to the National Rural and Remote Nursing Generalist Framework, which aims to understand the unique context of rural nursing and describe the key skills required (Office of the National Rural Health Commissioner, 2023). This framework also consists of four domains (culturally safe practice; critical analysis; relationships, partnerships, and collaboration; capability of practice), and outlines its application for registered nurses, education providers, and workforce development initiatives led by government stakeholders and relevant organisations (Office of the National Rural Health Commissioner, 2023).

### Clinical implications

#### Workforce development

As identified by participants, a novel rural psychology competency framework offers multi-dimensional utility across all levels of the rural pipeline model. Challenges with rural mental health service accessibility begin with service availability, with a limited number of psychologists practicing in rural areas (AIHW, 2024a). A model of rural psychology practice may be useful in promoting the profession to both psychologists and organisations. Additionally, psychologists considering a move to rural practice could be better informed about role and lifestyle expectations. Employers could also use such a model to assess which candidates may be best suited and prepared for rural roles, potentially supporting workforce retention. For example, Onnis (2019) reported that person-fit approaches to recruitment (i.e., matching a person’s attributes to the role required), as well as ensuring prospective employees had a realistic understanding of their role, improved retention amongst remote Australian health workers, suggesting the potential applicability of a rural psychology model in supporting current recruitment methods.

#### Training

Other strategies to encourage the retention of rural psychologists are to ensure they are adequately trained and supported to manage likely occupational and lifestyle challenges. Similar to other rural health frameworks (e.g., The National Rural Generalist Pathway; Worley et al., 2019), the current model provides a platform for developing and delivering rural-relevant training and professional development, at both an institutional and professional level. Given the limited number of rural psychologists, rural-based supervisors face their own challenges when offering supervisory support (Waugh et al., 2023), with many supervisees having to seek supervision from external urban-based professionals (Varela et al., 2021). Such supervisors can now consult the current model when supporting supervisees, ensuring a culturally and contextually relevant approach.

#### Self-reflection

For all psychologists, reflective practice is a vital skill, having recently been included in the updated competencies and professional standards required of psychologists in Australia (Psychology Board of Australia, 2024). Self-reflection using structured self-assessments can assist psychologists with goal setting and developing a broader understanding of their competencies (Banner et al., 2025). In the current study, participants identified the utility of the rural psychology practice model as a tool for self-reflection. Specifically, clinicians identified the model as useful to facilitate reflective practice regarding contextually relevant skills, consistent with the professional and ethical requirements of psychology practice. At a tertiary level, reflective practice is already integrated into accredited training programmes (Australian Psychology Accreditation Council, 2025). The current model aligns with current education approaches, supporting trainee and early-career psychologists interested in rural practice develop their reflective capacities. For rural psychologists at all career stages, supervision and professional support is often delivered remotely (Varela et al., 2021). Clinicians could use the rural psychology practice model to guide structured self-reflection between supervision sessions, identifying learning needs and areas for discussion, and potentially facilitating focused supervisory support.

#### Effective practice

The overall utility of the current model is underscored by its potential impact for clients in rural Australian communities. In recent updates to the competencies required of psychologists, the Psychology Board of Australia (2024) recognised culturally responsive practice as a key competency. Rural residents have reported that, although service accessibility is a major barrier to engaging with mental health services, available services also need to be culturally appropriate and relevant to the specific communities they serve, with a preference for in-person rural-based support (Moody et al., 2025, 2026a). Though there is currently no explicit definition pertaining to rural culture, broadly it is understood as the individual, sociological, historical, and economical influences on a community, and how these differ between rural and urban locations (Ragusa & Johnson, 2014). Clinicians working rurally should be aware of these influences and adapt their approaches specific to the communities they are supporting (Helbok, 2003). The model of rural psychology practice supports clinicians to understand the impacts of rurality more broadly, as well as how to adapt their clinical approaches specific to culturally responsive practice.

Should telehealth present as the only available modality, rural residents prefer that urban practitioners providing such services be trained in rural mental health, emphasising the importance of receiving culturally and contextually relevant care (Moody et al., 2025). These findings were echoed by the participants of the current study who acknowledged that a model was needed due to both the limited services available and the unique context of rural areas. Given geographic and workforce challenges, universal access to in-person support is unlikely to be feasible for some rural communities. Rather, the current model offers utility in preparing urban clinicians to provide appropriate telehealth, relevant to the rural culture. By training and supporting more psychologists to meet the mental health needs of rural people, both in-person and online, communities can receive safe, effective, and equitable mental health care services.

Unlike other rural health professions such as medicine and nursing (Office of the National Rural Health Commissioner, 2023; Worley et al., 2019), rural psychology is not yet recognised as a specialised role. Overall efforts to increase the rural psychology workforce are impeded by various structural barriers, including limited rural placements and predominantly urban-based training and supervision (Little et al., 2019; Varela et al., 2021). However, the current model, having been developed in the context of current psychology training and workforce infrastructure, is designed to support psychologists within these barriers. For example, its potential application across training, supervision and professional development opportunities, ensures the model can be incorporated into existing workforce approaches.

### Limitations and directions for future research

There were some limitations in the current study. Initial model development was primarily informed by the authors’ own research programme. Combined with a single-analyst approach to the broader methodology, this process introduces a potential for circularity. The integration of the wider evidence base into the model (e.g., Australian Psychological Society, 2016; Bischoff et al., 2014; Bourke et al., 2004; Helbok et al., 2006; Sutherland & Chur-Hansen, 2014), alongside reflexive research practices (e.g., reviewal of items with the wider research team, journalling and documentation of changes and steps), and refinement by way of expert review and the Delphi process aimed to mitigate against methodological limitations, and strengthen the validity of the model. Furthermore, items introduced in round 2, such as those corresponding with the digital adaptations domain, reached consensus consistent with the study protocol and did not undergo further review. Subsequently these items were not presented to the panel alongside aggregate feedback for reconsideration.

The sample size of 28 at Round 1 and 22 at Round 2, although considered acceptable (Chalmers & Armour, 2019), was smaller than the average size of Delphi studies commonly reported in health literature (Schifano & Niederberger, 2025). Participant demographics were limited to majority Caucasian, female, and living in inner regional Australia. Though a small number of participants were from outer regional locations, none were from remote areas, potentially limiting the relevancy of the model to the broader rural psychology workforce. However, location is not necessarily indicative of rural expertise, given that many rural and remote health services are delivered by a variety of modes, including outreach and telehealth services (Wakerman et al., 2008), and likely by clinicians from regional and urban locations. Rather, the sample may have been limited by purposive recruitment through rural-focused professional networks and favouring participants with a specific interest in rural psychology.

To this end, a limitation to the model itself may be in its utility to the rural psychology profession as a group, rather than to specific roles, such as fly-in-fly-out or telehealth roles. Due to the geographic spread of rural and regional communities, many health services rely on these unique roles to ensure that communities receive adequate support (Wakerman et al., 2008). Some competencies within the rural psychology practice model, such as digital adaptations, may be more relevant to some roles than others. However, as a novel framework, the development and refinement process considered all roles. Furthermore, the current iteration of the model is likely to evolve during its implementation and as the evidence-base expands. Future research should explore the model’s various avenues of utility discussed earlier in this paper, including its application in recruitment and retention, training, reflective practice, and improving the efficacy of mental health services in rural communities.

The challenges of mental health service delivery in rural and remote communities are not unique to Australia and an additional area for future research is the international applicability of the current model. For example, rural communities in both the United States and Canada are impacted by a combination of geographic limitations, sociocultural factors and shortage of healthcare providers, leading to poorer mental health outcomes compared to urban communities (Fleming & Sinnot, 2018; Friesen, 2019; Iglehart, 2018; Morales et al., 2020). Similar to Australia, rural mental health clinicians in the United States and Canada experience challenges related to multiple relationships, complex cases, and high workloads, while working with limited professional support (Gillespie & Redivo, 2012; Hastings & Cohn, 2013). Malone (2011) acknowledged the uniqueness of rural psychological practice in Canada, defining the role as generalist in nature, and requiring training distinct from current urban training practices. However, to our knowledge, our model is one of the first empirically validated models to describe the competencies and skills required for rural psychological practice. Though it is uniquely situated within the Australian rural context, there exists an opportunity for future research to modify and validate the current model for other contexts and improve mental health service access in rural communities internationally.

## Conclusion

Rural Australians need greater access to psychological services that are contextually and culturally relevant to their communities (Moody et al., 2025, 2026a). Rural psychologists require greater training and support in servicing such communities (Moody et al., 2026b, 2026c). The current study aimed to validate a novel framework that highlights and describes the skills and competencies required of a rural psychologist. Using a Delphi approach, rural psychologists and academics from across Australia achieved consensus on model items following two rounds of surveying and refinement. The resulting validated model of rural psychology practice described eight thematic competencies informed by an overarching understanding of the rural context. The model has applications in workforce development strategies, training and supervision, and may provide an opportunity to inform the development of a rural generalist specialisation in psychology. Overall, such a model contributes to the delivery of safe and effective mental health care in rural Australia.

## Data Availability

The data that support the findings of this study may be made available from the corresponding author upon reasonable request.
